# From village to state, and back again: cycles of de-urbanization and adaptation in Early Historic and Medieval Sri Lanka

**DOI:** 10.1080/00438243.2026.2664414

**Published:** 2026-05-01

**Authors:** Robin Coningham, Christopher Davis, Prishanta Gunawardhana, Mark Manuel

**Affiliations:** a Department of Archaeology, Durham University, Durham, UK; b Department of Archaeology, University of Kelaniya, Kelaniya, Sri Lanka

**Keywords:** De-urbanization, urbanization, collapse, persistence, mobility, Sri Lanka

## Abstract

Many have attempted to identify the causes of the abandonment of Sri Lanka’s first capital, Anuradhapura, but few have documented the characteristics of its de-urbanization. By mapping disruptions to established networks of resource procurement and production across its hinterland settlements, we present evidence that the city reverted to procuring and processing its own materials in its terminal phases, coming to resemble the footprint of a large village. Its successor, Polonnaruva, was recast as a centralized space in reaction to the challenges which had led to the demise of its predecessor. Despite accommodating new realities, it also proved unsustainable within the island’s Dry Zone. These experiments led to a distillation of courtly activities into a series of compact ceremonial centres which replicated a cosmological template as elites attempted to display continued legitimacy and hegemony within the more benign yet circumscribed environment of the island’s Intermediate and Wet Zones. This paper thus explores dynamic cycles of Sri Lankan urbanization, de-urbanization and re-urbanization over the timeframe of two millennia as communities attempted to mitigate both climatic and political stresses.

## Introduction

The abandoned cities of Sri Lanka’s Dry Zone (DZ) were marvels for nineteenth-century visitors as the ‘rediscovered’ ancient capitals of Anuradhapura and Polonnaruva were situated in sparsely populated malarial forests (Forbes 1840; Knighton 1845). The question of how these cities developed in the DZ’s challenging environment was answered by the identification of a system of reservoirs and canals running parallel to small, localized gravity-fed cascade tanks (Coningham and Lucero 2021). This innovation supported cities despite an annual rainfall of 1200–1900 millimetres, concentrated between October and February (Coningham 1999, 9). With evapotranspiration rates exceeding precipitation, early colonial records estimated that this region could sustain a population of some 0.4 individuals per square kilometre, increasing to over 200,000 people when supported by artificial irrigation (Coningham and Allchin 1995, 173). As water management was essential for concentrations of people, livestock and crops within the DZ, it is natural that it also influenced explanations for the emergence and decline of both Anuradhapura and Polonnaruva.

A general archaeological focus on ancient civilizational failure and ‘collapse’ has overshadowed explorations of persistence built on mitigation and adaptation (Crawford et al. 2023, 214–215; M. E. Smith et al. 2021). However, understanding how de-urbanization processes, including settlement contraction, de-centralization, and shifts to new centres, relate to these factors is invaluable, as lessons learnt from the past regarding sustainability, vulnerability and adaption are being increasingly recognized as having application in the present and future (Coningham and Lucero 2021).

## Invasions and the ‘catastrophic’ de-urbanization of the Dry Zone

Using textual and historical narratives, colonial administrators identified migrations and invasions as the explanation for the emergence and destruction of complex societies across South Asia, including Sri Lanka (Coningham et al. 2017, 23). Drawing from the island’s Pali Chronicles, officials used the *Mahavamsa*, *Dipavamsa* and *Culavamsa* to construct a historical framework of the island’s communities from the mid-first millennium BCE to European colonization. Compiled by Buddhist monks in the Early Historic and Medieval Periods (Geiger 1912 (1992), 1912 (2003)), these texts later became privileged sources for studying Sri Lanka’s past (Seneviratne 1997) and identified three pre-European communities, each with an origin narrative, which were mapped onto modern communities; the *Sinhala* as the island’s Buddhist population, the *Pulinda* as the contemporary hunter-gatherer groups known as the Vadda, and the *Damila* as the island’s Tamil communities (Coningham and Lewer 2000, 708). Both Anuradhapura’s and Polonnaruva’s decline were directly attributed to *Damila* and South Indian incursions, resulting in Anuradhapura’s destruction in the eleventh century CE (Strickland 2017, 1) and Polonnaruva’s in 1215CE (Indrapala 1971a, 73; Liyanagamage 1971). Although archaeological evidence was weak, and the Chronicles represented the narrative of only one of the island’s communities (Coningham et al. 2017, 23), invasions were often accepted as the main cause of de-urbanization, an explanation persisting into post-independent Sri Lanka (Seneviratna 1994, 34).

While invasions are often framed as leading to rapid collapse without recovery, this has been disputed at Anuradhapura. Several military incursions are recorded before the eleventh century CE (Paranavitana 1971, 50), and geoarchaeological evidence suggests a protracted decline with the continuance of small-scale populations and cultivation into the seventeenth century CE (Gilliland et al. 2013, 1026), alongside a continued veneration of sacred locations (Bailiff et al. 2013, 198). This supports the wider discourse surrounding definitions of collapse, which also points towards a drawn-out process where some elements of society disappear, with others continuing or transforming (Middleton 2025).

Rather than an unilineal event, several additional single and multicausal factors have been associated with de-urbanization within the DZ, including climate change (Gilliland et al. 2013; Paranavitana 1971); disease (Brohier 2006; Indrapala 1971b); administrative change (Davis et al. 2013; Indrapala 2005; Spencer 1983); and the lack of powerful leadership (Liyanagamage 1971; Paranavitana 1971). Despite this breadth of causes, the associated degrading of hydraulic infrastructure unites hypotheses as water was critical for the maintenance of large-scale populations and agriculture within the marginal landscape of Sri Lanka’s DZ.

In contrast to earlier narratives focused on catastrophic collapse, it is now possible to identify adaptations, mitigations and transformations that enabled these past communities to persist. Using examples of Anuradhapura and its successors, the responses to the various challenges faced will now be explored, identifying processes of urbanization, de-urbanization, mobility of the state and continuity of landscape use, as well as the persistence of ritual centres.

## The context for de-urbanization in the Dry Zone: persistence, adaptation and transformation

Anuradhapura presents a success of persistence over 1900 years, most of these as the island’s capital, which saw the settlement’s transformation from an Iron Age village in the eighth century BCE, to a cosmopolitan Medieval metropolis (Coningham 1999). Aligning to Early Historic urban planning treatises, such as the *Arthasastra* (Olivelle 2013, 103), the city of Anuradhapura was laid out by enclosing 100 hectares of cardinally orientated roads and structures within a quadrangular rampart and moat by the fourth century BCE (Coningham 1999). Known as ‘the Citadel’, it was surrounded by a ring of monumental reservoirs, constructed from the fifth century BCE through to the first century CE.

This outer ring hosted the ‘Sacred City’ of 2,500 hectares of Buddhist monasteries (Bandaranayake 1974). The Chronicles record that Buddhism arrived on the island in the third century BCE and was swiftly adopted by the kings of Anuradhapura (Mahavamsa 14.59–64). Royal legitimization was interlinked with the propagation of the Buddhist monastic order, or Sangha (B. L. Smith 1978a, 58–62; 1978b, 83), accompanied by the acquisition and deposition of relics at Anuradhapura, including the Buddha’s collar bone encased within the Thuparama Stupa (solid mound of stone or brick), and a cutting from the Bodhi tree at Bodh Gaya in northern India newly planted in the Sri Maha Bodhi complex (Mahavamsa 17 and 18; Frasch 2010, 648). These were followed by the construction of gigantic stupas within the complexes of the three monastic fraternities at Anuradhapura between the second century BCE and fourth century CE, the largest of which, Jetavana, measured 160 metres high and contained an estimated 200 million bricks (Coningham 2014).

Although the city physically followed the prescriptions of the *Arthasastra*, its hinterland did not reflect the recommended hierarchical model of settlement. Archaeological survey has identified a low-density dispersed agrarian urban settlement pattern and an absence of towns or a tiered settlement hierarchy (Coningham et al. 2013, 468). Forming a heterarchy, Buddhist monasteries administered autonomous domains, ‘temporalities’, and functioned as permanent centres in place of towns around which short-term shifting village settlements pivoted, as indicated archaeologically by shallow ceramic scatters (Coningham et al. 2007; Gilliland et al. 2013). Persisting from the Early Historic Period (340BCE–200CE) to the end of the Early Medieval Period (600–1200CE), this was not a static system, and dynamic changes occurred, particularly within the later phases of the hinterland’s occupation, demonstrating mitigations and adaptations to societal and environmental challenges.

It should also be recognized that population levels within these centres were transient and seasonal in nature even during major phases of occupation, leading to short-term cycles of urbanization and de-urbanization. At Anuradhapura, regular pilgrimages and festivals linked core with periphery, bringing rural communities into the centre and facilitating social and economic interactions (Coningham et al. 2013, 470). Likewise, the communal investment required for building Anuradhapura’s city ramparts, and the construction and maintenance of reservoirs and canals would have required additional labour, a premise that can be extended to the major monuments of Anuradhapura’s Sacred City.

Analysis of ‘Immunity Grant’ inscriptions suggests that rulers steadily transferred authority over labour and agricultural surplus to autonomous Buddhist monasteries across the hinterland from the ninth century CE onwards within the Early Medieval Period (Davis et al. 2013; Dias 2001, 113). Undertaken to maintain support and legitimacy at, and beyond, the limits of royal hegemony, this process may also have weakened and constrained centralized control over the hinterland, and particularly access to labour for annual irrigation maintenance (Davis, Coningham, and Gunawardhana 2025; Liyanarachchi 2009, 108). The gifting of immunities coincided with the Medieval Climate Anomaly (MCA) between the ninth and eleventh centuries CE. Associated with general warming and increased average rainfall, the MCA has been identified as beneficial for agricultural production and yields (Fletcher 2018, 247; Lieberman 2011). Thus, the MCA may have aided continued agricultural production and yields while centralized control and maintenance diminished.

A further climatic factor occurred at the end of this period in c.1100CE, with a high-amplitude increase of the southwest monsoon that precipitated cycles of severe droughts and intensified cyclones and associated flash-flooding within the northeast monsoon (Gilliland et al. 2013, 1026; Jung et al. 2004). In combination with the growing number of autonomous Buddhist institutions withholding annual labour and taxes through their immunities, this would have caused stress across the hydraulic infrastructure of Anuradhapura’s hinterland which could not be addressed due to the reduced capacity and reach of royal authority to maintain, repair, or construct the core irrigation infrastructure on which the wider system relied (Davis, Coningham, and Gunawardhana 2025). Increased climatic volatility, combined with this transfer of administration and increased political pressures from southern India stretched the agricultural and hydraulic systems, which began to exceed their in-built resilience and capabilities (Gilliland et al. 2013, 1026; Strickland et al. 2018, 276).

At the same time, archaeological evidence indicates the character of centralized and localized responses. The first was the establishment of *Pabbata Viharas* (700–1200CE), monasteries directly linked to royal patronage (Bandaranayake 1974, 81; Coningham et al. 2007, 709), as attempts were made to re-established centralized royal control in the hinterland to counterbalance the transfer of authority enacted by Immunity Grants (Davis, Coningham, and Gunawardhana 2025). They were accompanied by a corpus of small ‘focal’ stupas built on top of existing monumental stupas within the hinterland, again visibly reaffirming central patronage (Bailiff et al. 2013).

In contrast, Anuradhapura witnessed an increase in the cosmopolitanism of ritual foci (Coningham et al. 2017, 37) and growth of a network of non-Buddhist ritual sites across the landscape from the tenth century CE. Identified through caches of terracotta figurines and anthropomorphic phalli, their uniformity suggests a parallel ritual network to Buddhism (Coningham et al. 2012, 2). Figurines were deliberately broken and may be a forerunner of the *Gammaduva* ceremony, which includes the purposeful breaking of terracotta objects to ward away illness and ensure good luck, prosperity and fertility for people and agriculture (Coningham et al. 2012). This perhaps represents a response to decreased agricultural returns from a declining irrigation system and climatic pressures, where populations looked to other ritual and cosmological safeguards (Coningham et al. 2013, 468).

Despite these responses, most of the hinterland’s irrigation infrastructure fell into disuse between c. 1100–1200CE, as evidenced by siltation within channels (Gilliland et al. 2013, 1025). This also mirrors the end of large-scale occupation within the city (Coningham 1999) and its hinterland (Coningham et al. 2013, 467), where small-scale shifting cultivation, not dependent on artificial irrigation, continued (Gilliland et al. 2013, 1026). The hydraulic system became increasingly inflexible and vulnerable through path dependency and climatic instability, which alongside social and political pressures strained the embedded water systems of the DZ (Lucero, Fletcher, and Coningham 2015, 1148–1150; Strickland et al. 2018, 276–277).

## Mapping characteristics of de-urbanization at Anuradhapura

The repercussions of these changes for urban populations during the Early Medieval Period can be mapped archaeologically within Anuradhapura’s Citadel. Having recorded the presence and absence of datable layers across the Citadel through auger core profiles and excavation trenches, it is clear that Anuradhapura’s occupied size reduced from 100 hectares in the Early Historic Period to 70 hectares by the end of the Early Medieval Period (Coningham 1999, 20). This was accompanied by the disruption of established redistributive networks as indicated by changes in artefactual and material evidence on the premise that relative weight distribution within the excavated sequence may be indicators of change. This is particularly highlighted for comparisons between the Early Historic and Early Medieval Periods of Trench ASW2 (Coningham 1999), which have a similar volume of occupation within the sequence with c. 250 and 200 cubic metres, respectively, and served a similar residential function.

**Figure 1.**
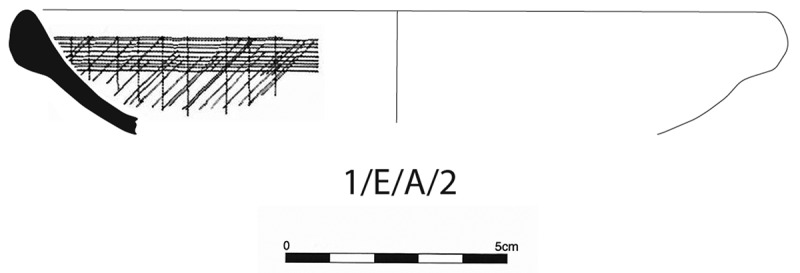
*Nambiliya* ceramic form (after Coningham (2006. Figures 6.12 and 6.27)).

**Figure 2.**
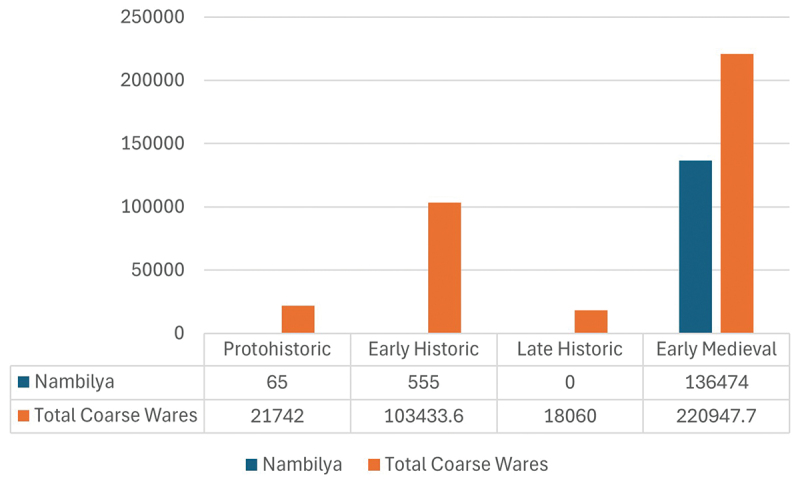
Weight (grams) of *Nambiliya* and total coarse wares within the ASW2 sequence by chronological phase.

**Figure 3.**
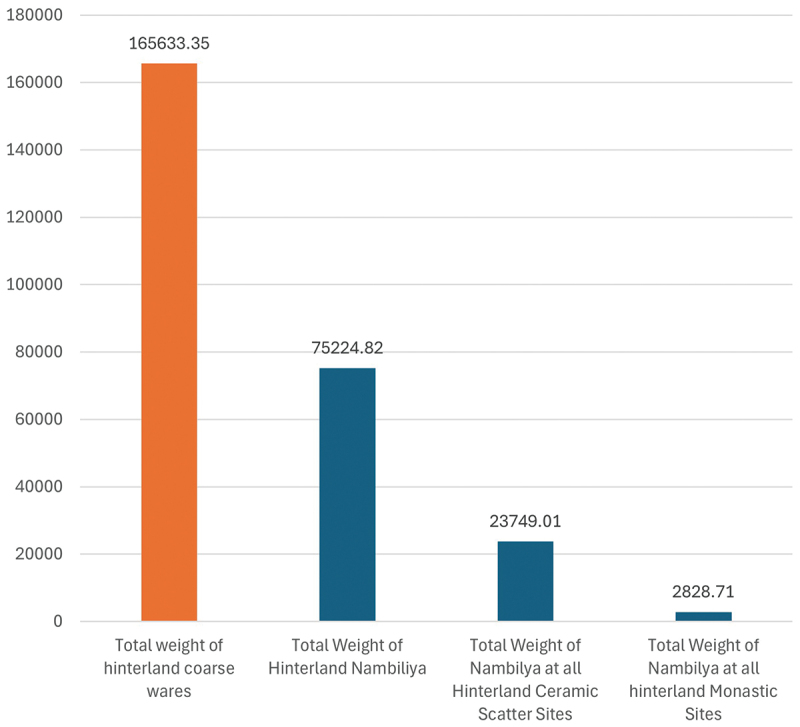
Weight (grams) of *Nambiliya* and total coarse wares recovered from the Anuradhapura hinterland.

One potential marker of change is a class of shallow bowls with wide mouths and an external rim found within Anuradhapura’s coarseware ceramic assemblage. Corresponding to the traditional Sri Lankan *nambiliya*, they are incised internally and this feature has been associated with food preparation and the separation of sand and grit from rice grains (Gunasekera, Prematilleke, and Silva 1971, 169) (Figure 1). With the premise that *nambiliya* indicates processing, it is understood that its presence can help identify whether settlements were locales for processing or receivers of processed resources. Within the city, there is a major shift in proportions of *nambiliya* from representing a negligeable component of the coarseware assemblage before the Early Medieval Period, to representing 61.76% of the weight of the entire corpus (Figure 2). This shift reflects disruption to the citadel’s traditional patterns of resource procurement and redistribution from the wider landscape, indicating increasing numbers of the urban community began processing their own rice. Within the hinterland, *nambiliya* provided 45.41% of the diagnostic coarseware assemblage, indicating the hinterland always undertook food processing for consumption as well as redistribution. Of these, 31.57% of *nambiliya* were found at ceramic scatter sites, whereas only 3.7% were found at sites with monastic occupation, reaffirming the role of monastic sites as administrative nodes in the hinterland, recipients of processed surplus and centres of redistribution (Coningham et al. 2007) (Figure 3).

When analysed by chronological phase, the relative presence of *nambiliya* at both monastic and ceramic scatter sites can also be used to infer levels of centralization across the hinterland. Found in high quantities at ceramic scatter sites, as well as at sites that would later become locations for monasteries, their distribution suggests that the hinterland was de-centralized with independent networks of production. The percentage of ceramic scatters with *nambiliya* remained substantial and increased slightly from the Early Historic period through the Late Historic and into the Early Medieval period suggesting that these sites continued in importance for food processing. This contrasts with monastic sites where *nambiliya* were present but found at just under a quarter of these sites during the Early Historic Period and Late Historic Period, indicating that these sites received surplus. The presence of *nambiliya* at monastic sites then increased in the Early Medieval Period, potentially indicating that food processing had again become more de-centralized across the entire hinterland and was being maintained within localized and independent networks (Table 1).

**Table 1.** Ceramic scatter sites and monastic sites within the Anuradhapura hinterland, including those with *Nambiliya*, by chronological phase.

Chronological Phase	Ceramic Scatters			Monastic Sites		
	Total Sites	Sites with *Nambiliya*	As a %age	Total Sites	Sites with *Nambiliya*	As a %age
Protohistoric	57	34	59.65	5	3	60.00
Early Historic	115	64	55.65	66	16	24.24
Late Historic	78	49	62.82	43	11	25.58
Early Medieval	233	155	66.52	44	19	43.18

**Figure 4.**
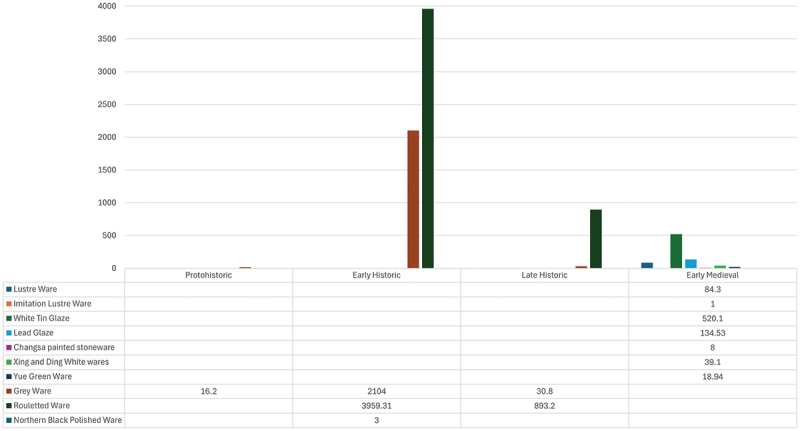
Weight (grams) of imported fine ware within the ASW2 sequence by chronological phase.

In parallel, imported fine ware at trench ASW2 decline from a peak of just over 6 kilograms in the Early Historic Period to 0.825.97 kilograms in the Early Medieval Period (Figure 4). Although fine wares were still imported to the Citadel, volumes decreased substantially and were also absent in the Early Medieval Period hinterland (Coningham et al. 2013, 467), further suggesting the weakening of bonds between the Citadel and hinterland.

**Figure 5.**
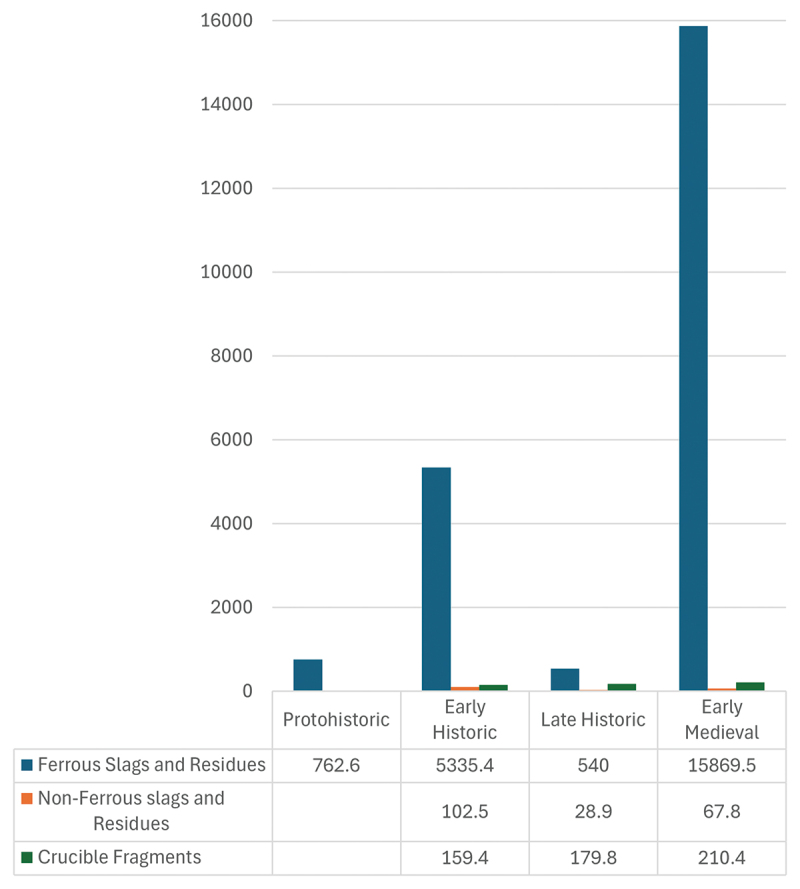
Weight (grams) of metalworking residue within the ASW2 sequence by chronological phase.

**Figure 6.**
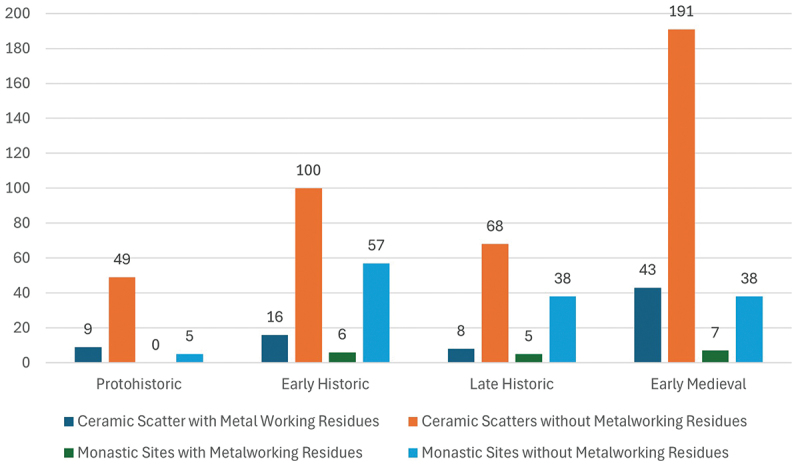
Weight (grams) of metal-working residues recovered from ceramic scatter sites and monastic sites in the Anuradhapura hinterland by chronological phase.

Patterns of metal-working also changed over time, with a modest presence within the Citadel, indicated by just over 5 kilograms of ferrous slags in the Early Historic Period, increasing threefold to almost 16 kilograms in the Early Medieval Period (Figure 5) indicating that the city’s communities began to play an increasing role in their own manufacturing. This contrasts to the hinterland where metal-working remained relatively stable (Figure 6).

Patterns linked to building material procurement also changed. Many Late Historic structures within Anuradhapura’s Citadel and Sacred City were timber, brick and tile constructions framed within carved stone pillars (Bandaranayake 1974). While gneiss outcrops are found within the vicinity of the city, survey within its hinterland confirmed the location of ten quarries, some complete with finished pillars ready to be transported as well as partially completed ones. Most were located between 13 and 32 kilometres from the Citadel, with only one 4.17 kilometres away.

Structural phases corresponding to the subsequent Early Medieval Period indicate a major shift in construction material procurement, with the systematic extraction and reuse of stone blocks, bricks and construction materials from earlier buildings across the Citadel (Coningham 1999, 80). Reclaimed material was used to construct an ashlar wall on the top of an expanded city rampart as well as for new residential structures (Coningham and Batt 1999, 129). This inability to procure stone from beyond the city’s walls suggests that networks for resource extraction across the hinterland were also impacted in a similar way to food processing, long-distance trade and metal-working. In effect, the residents of the city began to become another village – albeit a large one, within its own hinterland.

## The character of the city recast: Polonnaruva

One of the most significant aspects of Anuradhapura’s de-urbanization, and final adaptation to decline, was the permanent shift of authority to Polonnaruva (Frasch 2010, 653). Located 78 kilometres southeast and close to the Mahaweli Ganga, Sri Lanka’s longest river, Polonnaruva’s layout shared traits with Anuradhapura but was constructed on a massive scale. Polonnaruva was pre-planned with a citadel, inner city and outer city with walls, enclosing 200 hectares. It hosted numerous intra and extramural Hindu and Buddhist institutions. The incorporation of cosmopolitan communities and embrace of pluralism is often noted as a development of Polonnaruva but had already commenced at Anuradhapura from the Early Medieval Period (Coningham et al. 2017). Polonaruva was also connected to major irrigation works, and there was a continuity of artefactual and architectural style (Paranavitana 1955). The scale of the new city was massive, as the twelfth century CE tank, the Parakramabhu Samudra, covered 2,260 hectares, and the unfinished Damila Mahaseya Stupa was designed to be the island’s biggest with a diameter of 200 metres.

However, the ways in which the city and its hinterland were organized were markedly different from Anuradhapura, exhibiting centralizing tendencies. The religious complexes of the city and hinterland were pre-planned, indicating increased direct control (Bandaranayake 1989, 190; Manuel et al. 2021, 263–266; Paranavitana 1971, 81). Polonaruva was constructed with one pre-eminent monastic complex, the 80-hectare Alahana Parivena, which was physically and politically aligned closely with the royal line, avoiding earlier tensions between Anuradhapura’s three independent monasteries.

Polonnaruva’s hinterland was another example of low-density dispersed urbanism but demonstrates a top-down and centralized model when compared with the heterarchies surrounding Anuradhapura (Manuel et al. 2021). Ceramic scatters were denser in material culture, longer-lived, and larger in size in comparison with Anuradhapura. Only 1.1% of the 540 sites in Anuradhapura’s hinterland were over one hectare in size, clustering 22 to 28 kilometres from the Citadel, whereas 24.5% of the sites around Polonnaruva were over one hectare in size and were evenly distributed at a 5- to 30-kilometre distance from the centre, forming a clear hierarchy (Manuel et al. 2021, 257–258).

Together, these traits suggest that increased centralization at Polonnaurva was an attempt to avoid the weakened role of royal power that characterized the final phases of Anuradhapura. This is the scenario envisaged by Butzer and Endfield (2012, 3630), whereby the development of a negative feedback loop, as seen at Anuradhapura, leads to the emergence of stronger leaders with a reaffirmation of the cosmic and symbolic order, where ‘the primary kings of Polonnaruva sought not simply to keep alive the past but to establish a new ceremonial and political center to replace the old’ (B. L. Smith 1987, 77). In this model, political elites represent a focus of resilience and stability and, although they may not stop a cycle of decline, they can regenerate and revive dynastic ambition through the imposition of social and political structures at new political centres (Butzer 2012, 3637).

However, Polonnaruva’s centralization has also been cited a weakness. Although the concentration of power, with a peak during the reign of Parakramabahu I (r.1153-1186CE), may have increased efficiency, the state was more vulnerable during a crisis as there was less autonomy for decision-making (Paranavitana 1971, 55). It has been suggested that the strength of this centralized system was linked to the charisma of an individual and that when no longer at the centre, the system deteriorated (Liyanagamage 1971, 67). Unlike Anuradhapura, where de-centralization contributed to decline, there was too far a shift towards centralization at Polonnaruva.

As in the terminal phases of de-urbanization at Anuradhapura, stress to this system may also have come from additional political and economic factors, as well as climatic impacts such as the Little Ice Age (LIA) from the thirteenth century CE onwards. Indeed, the LIA has been identified as a factor in the decline of several complex societies in South and Southeast Asia. Rainfall and climatic assessments derived from tree-ring data and oxygen isotope composition analysis of speleothem suggest that they faced a series of severe ‘megadroughts’ and destructive floods through weaker and less predictable monsoons (Buckley et al. 2014; Fletcher 2018, 246–247; Lieberman 2011, 947; Sinha et al. 2011). Indeed, some of the basal infills of channels in Anuradhapura’s hinterland date to the thirteenth century CE (Gilliland et al. 2013, 1025), suggesting that communities which had persisted after the shift of capital finally abandoned the hydraulic infrastructure due to climatic degradation.

## De-urbanization and successive urbanizations: continuities and adaptations in the Intermediate and Wet Zones

Despite adaptations, the DZ state declined. Rather than a rapid collapse caused by invasions, multi-causal factors precipitated de-urbanization and population dispersal. Mitigations such as reformed administrative styles and diversified sacred safety nets failed to resolve the reliance on entrenched technologies and frameworks of large-scale irrigation systems, which forced a path dependency that was inflexible and un-adaptive to changing environmental pressures, as well as internal and external political factors (Coningham and Lucero 2021, 104). The low-density dispersed urban settlement pattern that had endured for over a millennium within the DZ became unsustainable.

The expansive low-density urban model was replaced by more compact urban expressions in previously peripheral areas, where new social, economic and political networks emerged in a process termed ‘Urban Diaspora’, mirrored in other tropical forest civilizations (Lucero, Fletcher, and Coningham 2015, 1139–1140). This involved the movement of population to the southwest and the Hill Country in Sri Lanka, corresponding with its Intermediate Zone (IZ) and Wet Zones (WZ). In contrast to the DZ, these areas receive between 1701 and 2201 millimetres of rainfall annually, and between 2201 and 4840 respectively (Roberts, Boivin, and Petraglia 2015). Despite abundant water throughout the year, the Hill Country had never been an intensively populated region (De Silva 2005, 134), even though it has early evidence of settlement (Deraniyagala 1992; Roberts, Boivin, and Petraglia 2015, 91). Until its transformation into the heartland of the island’s Late Medieval Period (c. 1200–1500CE) and its later capitals, it was viewed as ‘impenetrable tropical jungle … a sanctuary for rebels’ (Seneviratne 1978, 2). This is reflected in the concentration of inscriptions dating to the Early Historic Period, Late Historic Period and Early Medieval Period in the DZ (Davis 2018), despite over a century of systematic exploration in all the island’s zones.

Unlike the irrigation infrastructure of the DZ, agriculture within the IZ and WZ was rain-fed within bounded topography, focused on terraces with narrow dams across perennial rivers to irrigate crops. Some observers have interpreted this as a triumph of adaptation, the repurposing of existing technology to manage water in a new climate and geography (Brohier 2006, 54–56). Rice remained a major crop, but at a lower level of production (De Silva 2005, 119; Liyanagamage 1963, 228), with a switch to cultivating trading commodities, such as cinnamon and spices (Paranavitana 1971, 57).

**Figure 7.**
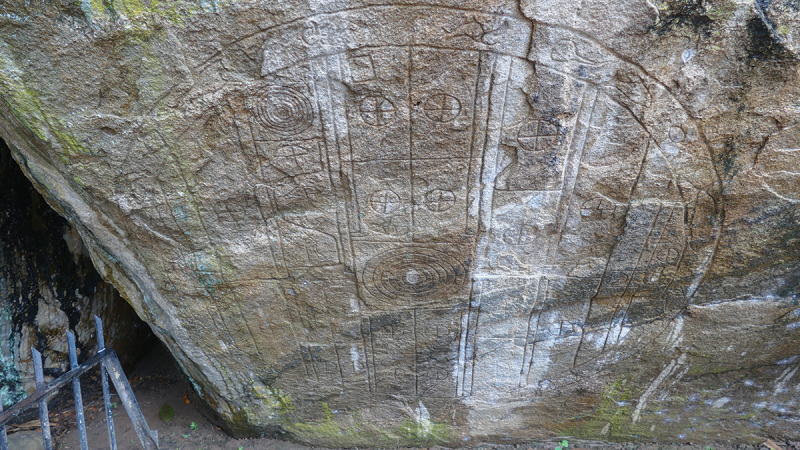
Cosmogram carved on a rock within the royal gardens of Anuradhapura (Authors’ Image).

While Anuradhapura and Polonnaruva were urban and ritual centres (Coningham 1999; Prematilleke 1982), they were also designed as cosmograms (Coningham et al. 2000), the moats and ramparts represent the ocean and mountain range that surrounded the universe, with a central royal palace representing Mount Meru, the dwelling of the gods (B. L. Smith 1987; Wheatley 1971) – an example of a carving of a cosmogram survives in Anuradhapura (Figure 7). Monarchs residing within palace complexes were locating themselves physically and centrally within society and cosmologically as *chakravartin* - universal rulers (Coningham and Lewer 2000; Wheatley 1971).

**Figure 8.**
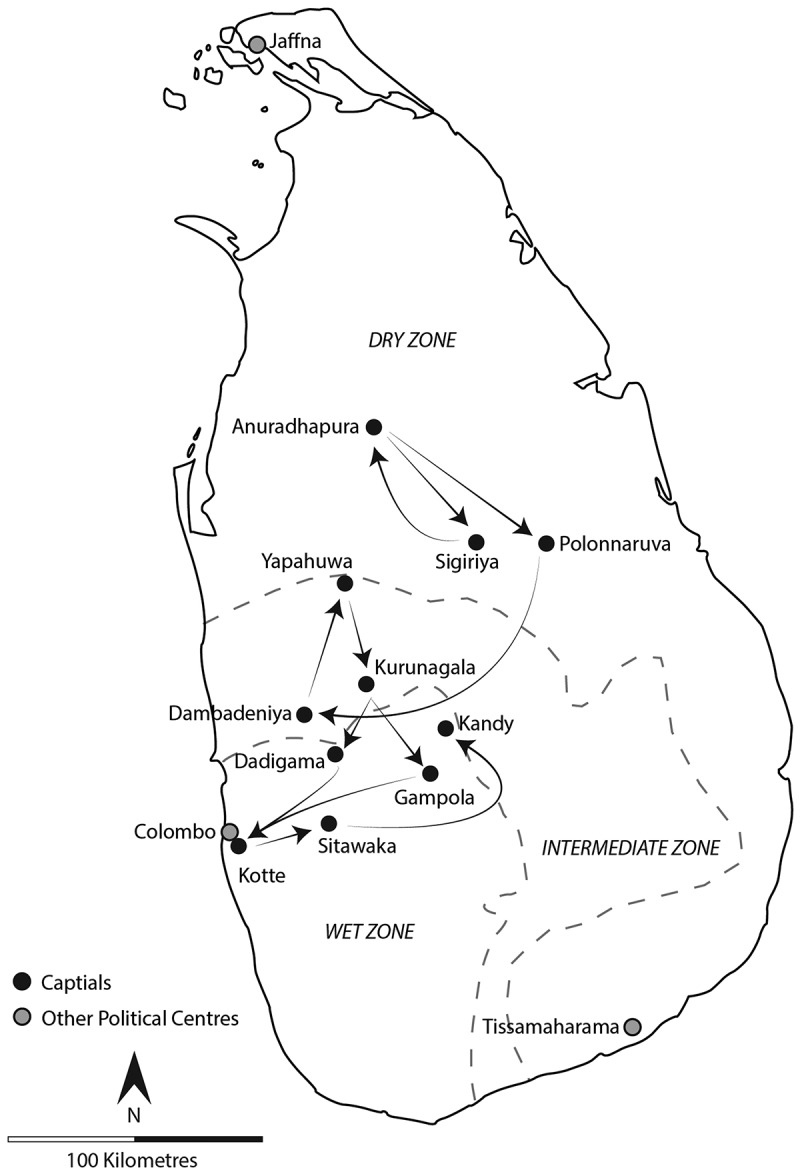
Movement of capital within Sri Lanka.

After the abandonment of Anuradhapura and Polonnaruva, a series of successor capitals emerged (Figure 8), many of which were constructed around rock outcrops. Viewed as defensive (Liyanagamage 1963: 196, 223), they also incorporated cosmological motifs, creating compact ceremonial complexes. A prototype had already been created within the DZ during Anuradhapura’s fluorescence at Sigiriya. Constructed 70 kilometres from Anuradhapura by the usurper Kassapa I (r.473-491CE), Sigiriya is interpreted as an attempt to recreate Alakamanda, celestial home of Kubera, god of wealth (Paranavitana 1950, 137). Sigiriya was designed with a walled outer zone with a tank, interpreted as reflecting the celestial lake Anottatta; the whitewashed boulders of the walled inner zone representing the Himalayas; and the citadel-palace itself, located on the summit of a 182 metre high outcrop to represent Mount Meru, likening the royal palace with that of Kubera. Despite adopting a symbolic layout, this experiment at projecting power and legitimizing authority did not outlive the usurper’s rule when he died at the hands of his brother 18 years later (Coningham et al. 2000). The return of the throne to Anuradhapura re-established its position as the sacred and ritual centre of the island. This was augmented by the development of a new and mobile symbol of ritual authority represented by the Buddha’s Tooth Relic. According to tradition, it arrived in the fourth century CE, and gained pre-eminence from the eleventh century CE onwards as the island’s symbol of sovereignty (Herath 1994, 93).

The later capitals lacked concentration of populations, either within their walls or hinterlands, and have been classed as ‘ceremonial complexes’ for ‘enacting the sacred through rite, symbol and cosmology’ (Bardwell B. L. Smith 1987, 75). While additional research is required to confirm their sequences, it is possible to identify a common set of key architectural attributes that accompanied the frequent shift of power.

The first of this series was Dambadeniya, founded in 1232CE (Culavamsa 81.15–16). Although its plan is ephemeral, the outer settlement was defined by three boundary walls with a further inner-city wall encircling a palace complex and Tooth Temple. An additional royal palace and Tooth Relic shrine sat on top of a large outcrop (Hocart 1928, 152). Dambadeniya was followed by Yapahuwa, which formalized into a political centre in 1270CE (Culavamsa 88.61 and 90.39). The outer settlement was defined by a moat and rampart, with gateways at the east, west and south, with the rock outcrop forming the northern boundary. A second rampart and moat defined the inner city. A monumental staircase rose 30 metres to an artificial terrace on the outcrop where the royal residence was located (Hocart 1928). The capital then moved to Kurunagala shortly after 1293CE (Culavamsa 90.59–64). The outer settlement was laid out as a cardinally orientated rectangle defined by moat and rampart, with the inner settlement delineated by an earthen rampart. The inner settlement included religious structures, including a potential Tooth Relic shrine, with the outcrop housing the royal citadel (Hocart 1928, 153).

The capital then moved into the Hill Country, with a system of joint kingship at Gampola and Dadigama, located 48 kilometres apart, with co-ruler brothers Bhuvanekabau IV (r.1341-1351CE) and Parakramabahu V (r.1344–1359CE), followed by Parakramabahu V and Vikramabahu III (r.1357-1374CE) (De Silva 2005, 114). Little remains of Gampola, although Hocart noted that the remains were identifiable within a tea estate, with a ‘secret refuge on the rock above’ (Hocart 1928, 153). Similarly, Dadigama is poorly preserved, but the *Tisara-sandesaya*, one of the Sinhalese *sandesa* (message) poems which were produced from the fourteenth to nineteenth centuries CE (Pieris 2010: 345), describes the site as the abode of the god Vishnu (Godakumbura 1968, 116).

The next move was to Kotte on the west coast, which represents the first instance of a successor city not constructed around an outcrop, and a reversion to the urban forms of Anuradhapura and Polonnaruva. It was defined by a river, lakes and marshes to the north, east and west, bolstered by laterite walls, bastions and gateways (Godakumbara 1976, 13; Wijesuriya 1986, 280–281). The size of Kotte was similar to the DZ capitals and coincides with the terminal phase of the severe megadroughts identified within the LIA in South Asia in the late fifteenth century CE (Sinha et al. 2011). Potentially, the regularity of monsoon patterns facilitated the stability of populations to agglomerate around an urban, rather than purely ceremonial complex and to harness the resources of its hinterland. Its coastal location also benefitted from the increasing demands on the island’s products from Indian Ocean traders.

Through pressure from regional kingdoms in continental India and European incursions from 1505CE, Kotte fractured with the development of a dynasty based at Sitawaka as the last king of Kotte bequeathed his kingdom to the Portuguese. Little remains of Sitawaka and the palace was said to have been destroyed by the Portuguese (Davy 1821, 264). This precipitated a move into the interior with the development of Kandy as capital. The last pre-colonial capital, it too can be read as a symbolic layout (Duncan 1990). To the east of the city, the palace and Tooth Relic temple were enclosed by a moat and a wall, which was decorated to represent the clouds around Mount Meru, while a lake was constructed to the south named after the Ocean of Milk creation story. Symbolically placing the King at the centre of the universe, further imagery placed him at the centre of the physical realm. Originally laid out as sixteen blocks, Sri Vikrama Rajasinha (r.1798-1815CE) elaborated this to twenty-one to mirror the kingdom’s twenty-one divisions (Duncan 1990, 93–94).

## Shared traits and mobile symbols of power

**Figure 9.**
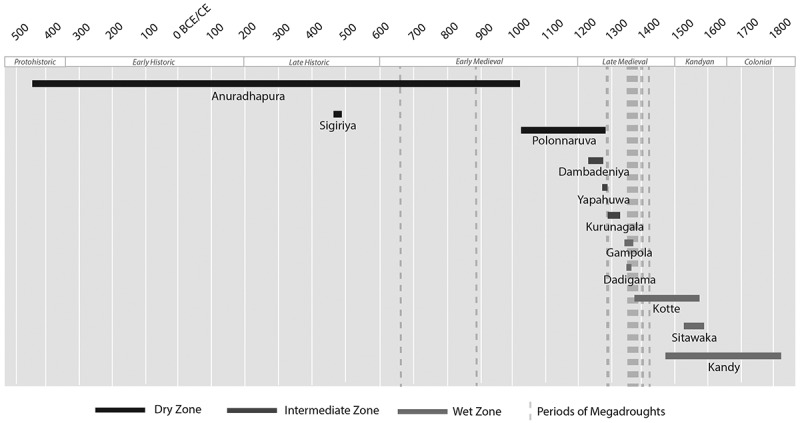
Longevity of sites as political centres in Sri Lanka, including location by climatic zone, compared with periods of megadrought identified by Sinha et al. (2011).

**Figure 10.**
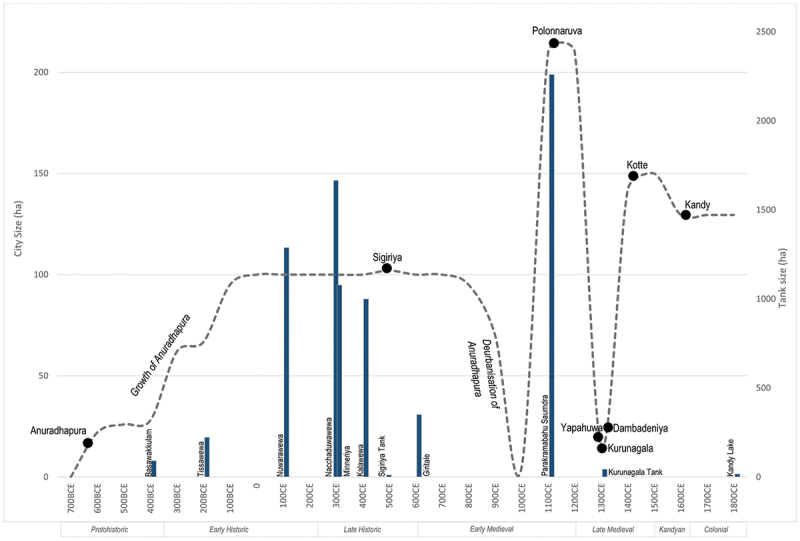
Size of enclosed area, where known, of political centre, and size, by surface area, of major tank constructions. Tank size represented by solid blue line with approximate size of enclosed areas of capital cities shown by dashed line.

The later diminutive capitals within the southwest lacked the ability to harness resource levels that could be attained from artificial irrigation infrastructure in the DZ. During times of climate instability and potential megadroughts, we suggest that alongside external and internal pressures, these sites could not achieve the massive scales and agglomerations of previous populations, constricting in size during the fourteenth to sixteenth centuries CE. They too could not be maintained for long-periods, leading to the short-term urbanizations and de-urbanizations of ceremonial centres, with larger and longer lasting capitals emerging again only after the megadroughts of the LIA (Figures 9 and 10). However, these sites shared a similar package of symbolic motifs, including outcrop palaces identified as Mount Meru or other residences of the gods, and walls, moats and lakes to delimit and represent the bounds of the universe. The idea of the continuity of the state rested on the repeated creation of urban forms that symbolically recreated the universe in microcosm to represent the monarch, state and the relationship of those residing within the realm. This repetitive pattern has been recognized elsewhere, with Bronson referring to it as ‘template regeneration’ (Bronson 2006, 140), whereby ‘reading, writing and record keeping permitted the reemergent state to copy the institutions and cultural styles of their predecessors with high fidelity and essentially re-create the past’ (ibid.: 217). Despite the de-urbanization and the de-population of the DZ, the royal line and its physical characteristics exhibited continuity and resilience, as identified across many ancient societies (Nicholl and Zerboni 2020).

Enabling this movement, the symbol of sovereignty of Sinhalese monarchs also became less geographically fixed. Unlike earlier immoveable relic stupas, the Tooth Relic could be transported with a monarch and court. Consequently, a Tooth Relic shrine was constructed close to the palace at each capital, from Anuradhapura onwards, demonstrating the link between spiritual and temporal authority. Through rituals and processions linked to the Tooth Relic, monarchs could demonstrate continuity of historical tradition and the order of society. Furthermore, as the relic was originally housed in Anuradhapura, it has been suggested that the sacredness of that centre was transferred to new capitals (Frasch 2010, 655–656, 661).

This sacredness continued to be embedded in the landscape and as mentioned above, the DZ was not entirely abandoned after the initial movements of capitals within and away from this region. Whilst Polonnaruva was developed as the new capital, chronicles record that Polonnaruva’s rulers undertook the restoration of monuments within the Sacred City of Anuradhapura (Strickland 2017, 8). Furthermore, small-scale cultivation continued in the DZ after the de-urbanization of Polonnaruva through to the recolonization of the region in the nineteenth century (Gilliland et al. 2013). During the reign of the Nayakkar Kings, who ruled from eighteenth and nineteenth-century Kandy, the DZ was under the influence of chieftains known as Vanniyas, who were also guardians of the Sri Maha Bodhi tree shrine (Sivasundaram 2013, 142, 144). The Nayakkars undertook pilgrimage to, and renovations of, Anuradhapura’s monuments and such acts affirmed their links to this sacred location to bolster their legitimacy as a royal line originating from outside the island (139).

## Conclusion

Additional excavation and hinterland survey at the successor capitals of Anuradhapura are required to further refine the models of urbanization, de-urbanization and re-urbanization presented. It must also be noted that while this paper has focused on the lineage that emerged at Anuradhapura, and continued with several iterations of dynasties through to Kandy, other kingdoms and powers were present on the island, such as the Cholas from the tenth century CE (Indrapala 2005) and later medieval regional polities, including the Jaffna kingdom (Rasanayagam 1926) prior to colonial contact. However, this paper has explored two crucial aspects of de-urbanization; firstly, the physical material evidence of the processes associated with the de-urbanization of the city of Anuradhapura and, secondly, the reoccurring cycles of re-urbanization and de-urbanization which followed its demise, providing an almost unparalleled sequence from the fifth century BCE to the nineteenth century CE.

While acknowledging that patterns derived from a single site may be inherently culturally specific, some of the patterns apparent in the physical material evidence for the processes associated with the de-urbanization of Anuradhapura are of broader significance. The pioneers of Anuradhapura designed an urban form which thrived as the island’s capital for almost one and a half millennia despite its challenging environment. Sharing distinct similarities with contemporary cities within lowland tropical forest belts, low-density urban form supported a dispersed population, coming together seasonally for ritual and labour at the centre (Lucero, Fletcher, and Coningham 2015). Once under pressure from combinations of factors, the processes of de-urbanization become apparent as the networks of urban procurement and consumption established for centuries were disrupted. Markedly, the city’s access to resources from its hinterland declined as its inhabitants began salvaging materials from earlier structures, processing their own rice and making their own tools. In effect, de-urbanization heralded a process by which the city became a village, albeit on a much larger scale, almost marking a ‘ruralisation’ (Yoffee 2005, 60), increasingly starved of material, surplus and labour.

Anuradhapura’s demise led to cycles of re-urbanization and de-urbanization. Anuradhapura’s low-density urban model and its complex hydraulic infrastructure were unsustainable due to its path dependency in the face of changing variables (Coningham and Lucero 2021). As a result, a new capital was established at Polonnaruva, offering an opportunity to recast the city and kingdom through new and cosmopolitan political, social and economic frameworks. Although elements of low-density urbanism, as well as architectural, technological and cultural continuity, are apparent, the city and its hinterland were highly centralized, seeking to avoid the eventual decentralization of Anuradhapura, which had generated destabilizing centrifugal forces. However, the city remained capital for little over 200 years and its de-urbanization led to the shift of sovereignty to the southwest in restrictive topographical and environmental conditions with reduced access to population, labour and agricultural surplus, these capitals became a compact form, representing a ‘ceremonial complex’ (B. L. Smith 1987, 75). Based on shared cosmological concepts, short-lived courts vied for hegemony engaging in ‘template regeneration’ (Bronson 2006, 150) to express legitimacy, enabled by possession of the Tooth Relic.

Reflecting on DZ de-urbanization, we should also note that later colonial administrators and engineers reanimated the dormant irrigation systems of the region in the nineteenth century at speed to re-urbanize the landscape, a process which continued throughout the twentieth century. Today the DZ’s population centres are clustered around these revitalized irrigation works, underpinning the success of its ancient technology. Indeed, the UN’s Food Agency Organisation inscribed the cascade tank system of the DZ on its list of Globally Important Agricultural Heritage Systems noting that it ‘has many beneficial characteristics such as expansive coverage, unique technology, sustainability, resilience to natural disasters and high biodiversity’ (FAO 2017). In line with the recommendations of UNESCO-ICOMOS-IUCN Co-Sponsored Meeting on Culture, Heritage and Climate Change, we recognize and repeat that ‘ … archaeology and heritage science, although infrequently mobilized, are uniquely placed to assist in providing a fuller understanding of the impact of climate change on urban infrastructure in the past; they also facilitate reflection on lessons of adaptation and resilience for modern cities and their inhabitants’ (Morel et al. 2022, 34).
